# Contemporary decongestant practices of Canadian otolaryngologists for endoscopic sinus surgery

**DOI:** 10.1186/s40463-019-0337-8

**Published:** 2019-03-18

**Authors:** Jonathan W. Reid, Brian W. Rotenberg, Leigh J Sowerby

**Affiliations:** 1 Department of Otolaryngology—Head and Neck Surgery, Western University, London, Ontario Canada; 2 Schulich School of Medicine and Dentistry, 1151 Richmond St, London, ON N6A 5C1 Canada; 3 St. Joseph’s Hospital, 268 Grosvenor Street, London, ON N6A 4V2 Canada

**Keywords:** Endoscopic sinus surgery, Decongestant, Cocaine, Epinephrine, Moffett’s solution, Practice patterns, Survey, Cardiac event

## Abstract

**Background:**

Cocaine has traditionally been the topical decongestant most frequently used for visualization of the surgical field in Endoscopic Sinus Surgery (ESS). Alternatives include xylometazoline, oxymetazoline, and epinephrine. The understanding of the safety profile of each agent is changing, as are the practices of Otolaryngologists-Head & Neck Surgeons. The objective of this study is to determine decongestant use practices in ESS across Canada, which has not previously been studied.

**Methods:**

A cross-sectional survey design using a 24-item electronic questionnaire was distributed to actively practicing members of the Canadian Society of Otolaryngology-Head and Neck Surgery via email. A French translated version of the survey was also available. Questions explored the respondents’ demographics and decongestion practices for ESS.

**Results:**

Ninety-six surveys from otolaryngologists practicing in Canada were completed (19% response rate). The average time in practice was 16.5 years (range 1–50 years, SD 12.0 years). Twenty-six (27%) of respondents use some form of cocaine solution for topical decongestion in ESS. Over a total of over 1500 combined practice-years, eight respondents (8%) personally experienced an adverse event that could be attributed to cocaine, including two mortalities. One cardiac even was directly attributable to the patients’ use of recreational cocaine in the immediate pre-operative period.

**Conclusion:**

The popularity of cocaine for topical decongestion in ESS in present-day Canada is less than in surveys from other countries. However, there are few reported adverse events with long-term consequences that are attributable to intraoperative cocaine. Considering the beneficial effects of cocaine for visualization and pain control, this change in practice warrants further investigation.

**Electronic supplementary material:**

The online version of this article (10.1186/s40463-019-0337-8) contains supplementary material, which is available to authorized users.

## Background

Endoscopic Sinus Surgery (ESS) is a common procedure, widely regarded as the standard of care for chronic rhinosinusitis refractory to medical management. Decongestant medications minimize the engorgement of the nasal mucosa and facilitate hemostasis, which is crucial to optimizing visualization in nasal surgery. For over a century, the most popular drug for this purpose has been cocaine.

Cocaine (benzoylmethylecgonine) is an alkaloid ester that provides topical analgesia, decongestion and hemostasis [1]. These properties make it valuable for nasal surgery. Cocaine has mostly been shown to be a safe agent, although rare serious idiosyncratic effects have been reported, even at a low dose [2]. The availability of cocaine has been decreasing because of its legal status as a controlled substance and the subsequent implications for procurement and storage. Thus, alternatives such as xylometazoline, oxymetazoline and epinephrine are increasing in use. Some of these alternatives have been demonstrated to be roughly equivalent to cocaine for decongestion, and may be especially appropriate when the patient has risk factors that could make the use of cocaine unsafe [3]. However, some cocaine alternatives, such as phenylephrine, also have significant safety considerations and have been implicated in perioperative adverse events [4]. A combination of cocaine and epinephrine (“Moffett’s Solution”) is sometimes used in preparation for endoscopic sinus surgery, as there is evidence that epinephrine reduces the systemic absorption of cocaine [5] and improves the surgical field [2].

A survey of American otolaryngologist – head and neck surgeons in 1977 found that 94% used cocaine in their procedures regularly, 35% use epinephrine with cocaine, and that 48% did not adhere to the recommended maximum dose of 200 mg of cocaine per patient [6]. A repeat survey 25 years later found that 88% had ever administered cocaine for nasal surgery, 50% had used it in the past year and only 8% had ever exceeded the 200 mg dose [7]. A 2002 survey of Australian otolaryngologists found 64% regularly administer cocaine in nasal surgery [1] and a 2003 survey of UK otolaryngologists and found that a total of 77% used cocaine in their nasal surgery patients, and 66% used a combination of cocaine and epinephrine [8]. There has never been a Canadian, nor an international survey of otolaryngologists’ practice patterns regarding cocaine for sinus surgery. The most recent questionnaire regarding decongestants in nasal surgery investigated practice patterns regarding epinephrine for ESS in 2011. The intent of the current study was to therefore describe the current practice patterns of Canadian otolaryngologists for decongesting the nose in ESS and also inquire about self-reported safety events noted by respondents.

## Methods

An online questionnaire methodology was developed in consultation with the Heath Sciences Research Ethics Board for Western University (London, ON – HSREB# 110291). An email list of active members of the Canadian Society of Otolaryngology—Head and Neck Surgery (CSOHNS) was used to distribute the questionnaire in both English and French. Each member received by email a notification including an introductory note describing the nature of the study and a hyperlink to a short anonymous survey using the Qualtrics platform. A single reminder email was sent 6 weeks later. The survey remained open over 8 weeks. Participation was entirely voluntary. To satisfy the inclusion criteria, participants must have been practicing staff surgeons and active members of the CSOHNS. Potential participants in residency or fellowship training were excluded as they would not yet make the ultimate decision regarding which decongestant preparation would be used. Only responses from those who completed the survey in its entirety were used.

The survey used a closed-question format asking about the physician’s use of cocaine and other decongestant drugs, their practices regarding local epinephrine injection, and adverse outcomes experienced. When necessary, questions included a space for comments. The survey included demographic questions about number of years in practice and practice type and location, as well as subspecialty training and training location. No compensation was offered to the participants, and no attempt was made to contact participants who did not return surveys. The survey is available online as Additional file 1 and Additional file 2.

The responses to each question were analyzed as proportions. The responses of different demographic groups were compared using Chi-squared tests. Short open text responses were analyzed descriptively. Descriptions of adverse events were screened for duplicate descriptions of the same event, using a combination of event descriptions themselves as well as demographic information.

## Results

**Fig. 1:**
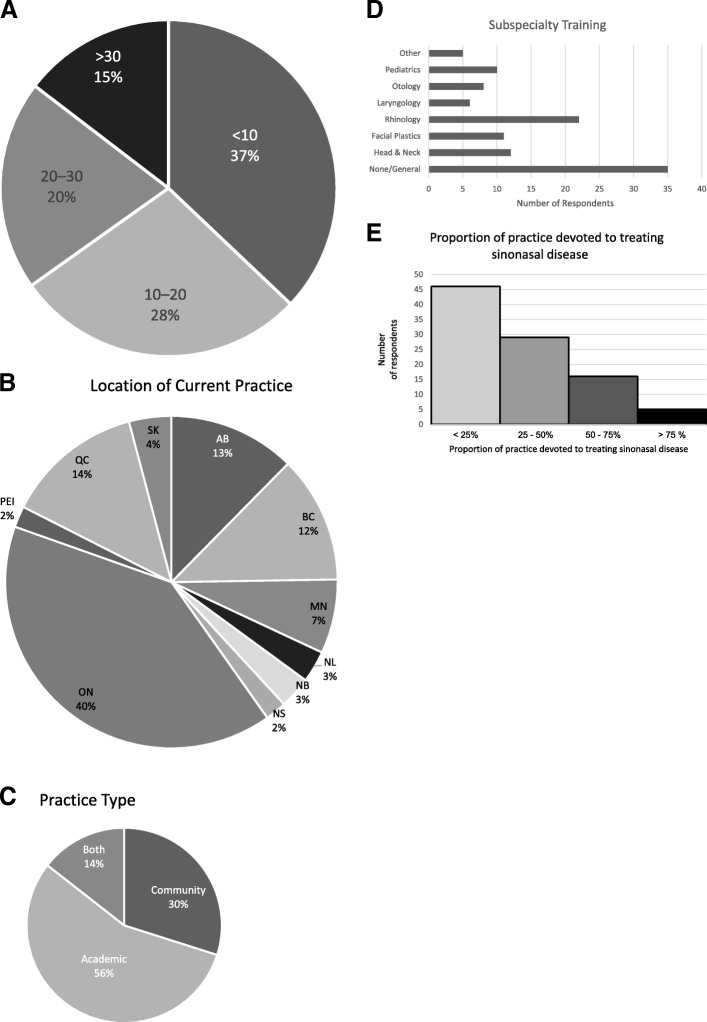
**a**-**e**: Epidemiological Data of respondents. Total *n* = 96

Ninety-six eligible otolaryngologists currently practicing in Canada returned the survey, representing a 19% response rate among eligible participants. Physicians from all 10 provinces responded. The average practice years per respondent was 16.5 with a standard deviation of 12.1 years and a range from 1 to 50 years. Figure 1 highlights the demographic data reported by respondents.

Twenty-two respondents (23%) used cocaine for decongestion in ESS. Six respondents (6%) used Moffett’s Solution (cocaine + epinephrine), including 2 respondents (2%) who used both Moffett’s and isolated cocaine, giving a total of 26 respondents (27%) who used a cocaine preparation in ESS.

Only 2 respondents reported using cocaine in pediatric patients, which represents 8% of the group of respondents who used cocaine. The other 24 respondents of this group (92% of respondents who use cocaine) reported that they avoided cocaine in pediatric patients.

Reported usage among the respondents is given in Table 1. Physicians who had been in practice for less than 10 years were less likely to administer cocaine than those who had been in practice for more than 10 years (32% vs. 12% [*P =* .037]). Cocaine usage also differed by province (Table 2), with the highest rate in Saskatchewan (4 of 4 respondents, 100% of Saskatchewan respondents), and lowest in Prince Edward Island and Nova Scotia (0 of 2 respondents in each case.) There was a similar proportion of cocaine usage between respondents who were fellowship-trained in rhinology (23%) and those who were not fellowship trained (28%) [*P* = 0.27] (Table 3). There was no significant association of proportion of cocaine usage with percentage of practice devoted to treatment of sinonasal disease (Table 4).

**Table 1:** Decongestants used by the respondent in ESS. Many respondents use more than one agent

	Number of Respondents^a^	Percentage of Total
Epinephrine (any preparation)	63	66%
Epinephrine (isolated)	60	63%
Xylometazoline	44	46%
Oxymetazoline	35	36%
Cocaine (any preparation)	26	27%
Cocaine (isolated)	22	23%
Moffett’s Solution	6	6%
Other	4	4%
Phenylephrine	1	1%

^a^Respondents who use Moffett’s solution (Cocaine + Epinephrine) were counted in the rows “Cocaine (any preparation)” and “Epinephrine (any preparation)”, respectively, with redundancies subtracted. One respondent responding “Other” commented that they do not perform ESS

**Table 2:** Provincial differences in cocaine and epinephrine use for ESS. Reported as percent of total respondents from that province

	Respondents who primarily use cocaine for ESS (any prep.)	Respondents who primarily use epinephrine for ESS	Respondents who only use other decongestants for ESS
	n, (%)	n, (%)	n, (%)
Alberta	2 (18%)	7 (64%)	2 (18%)
British Columbia	3 (25%)	2 (17%)	7 (58%)
Manitoba	3 (43%)	3 (43%)	1 (14%)
Newfoundland	0	3 (100%)	0
New Brunswick	1 (33%)	2 (67%)	0
Nova Scotia	0	2 (100%)	0
Ontario	9 (23%)	26 (67%)	4 (10%)
Prince Edward Island	0	2 (100%)	0
Quebec	4 (31%)	4 (31%)	5 (38%)
Saskatchewan	4 (100%)	0	0
Canada (Total)	26 (27%)	51 (53%)	19 (20%)

**Table 3:** Comparison of cocaine usage between respondents with rhinology fellowship training and respondents without rhinology fellowship training

	Rhinology Fellowship Trained	No Rhinology Fellowship	*P*-Value
Total number of respondents	22	74	
Respondents who use cocaine for ESS (any preparation)	5 (23%)	21 (28%)	0.27 (NS)

**Table 4:** Comparison of cocaine usage based on proportion of practice dedicated to sinonasal disease

	Proportion of practice devoted to sinonasal disease			
	0–25%	25–50%	50–75%	75–100%
Total number of respondents	46	29	16	5
Respondents who use cocaine for ESS (any preparation)	9 (20%)	11 (38%)	5 (31%)	1 (20%)

Within the group of respondents that utilized isolated cocaine, 19 respondents (73%) used a 4% solution, and 3 respondents (12%) used a 10% solution. Among users of Moffett’s solution, 2 respondents (33%) used 1.2% cocaine with 1:10000 epinephrine, 2 respondents (33%) used 4% cocaine with 1:1000 epinephrine, 2 respondents (33%) used 4% cocaine with 1:10000 epinephrine, and 1 respondent (18%) also used 10% cocaine and 1:10000 epinephrine.

Of those that used some form of cocaine in ESS, 14 respondents (54%) reported that their decision to use the agent was affected by the patient’s comorbidities, while 12 respondents (46%) did not change their use of the agent based on comorbidities. Some of the comorbidities respondents considered include allergy, coronary artery disease, arrhythmia, hypertension, and history of drug abuse. Only 2 respondents reported that they did not restrict dose of cocaine to 200 mg. Five respondents were unsure whether they dosed below 200 mg.

Of all respondents who used cocaine, 4 (15%) indicated that they personally experienced at least one adverse event that could be attributable to cocaine, in a combined total of 467 practice-years (Table 5). These included ventricular tachycardia converted without incident and hypertension. However, no mortalities were reported by those that use cocaine as an intra-operative decongestant. Among respondents that did not use cocaine, who share a combined 1063 total practice-years, 4 (6%) personally experienced at least one adverse event that could be attributable to intraoperative cocaine at some point in their careers. These included hypertension and cardiac arrest with recovery, as well as two mortalities: one caused by Torsades des Pointes and the other not specified. Although more serious adverse events were reported by the group that does not use cocaine for ESS, neither the average number of practice years per respondent nor the proportion of respondents who had witnessed an adverse event were significant between the two groups. Interestingly, two respondents reported an intraoperative cardiac arrest upon administering xylometazoline to a patient who had admitted to using cocaine before his procedure.

**Table 5:** Comparison of adverse events experienced among respondents

	Those who use cocaine for ESS	Those who do not use cocaine for ESS
Total number of respondents	26	70
Total practice years	467	1063
Mean practice years per respondent	20.3	15.2
		(*p* = 0.08, N.S.)
Experienced adverse event, possibly related to cocaine: n, (%)	4 (15%)	4 (6%)
		(*p =* 0.12, N.S.)
Types of adverse events	• Minor reversible arrhythmia • Hypertension	• Two mortalities • Hypertension • Arrhythmia • Allergic reaction

Among the 70 respondents who did not use cocaine for ESS, the most common reason cited was satisfaction with equivalent decongestants (46 respondents, 65%), followed by the poor availability of cocaine (30 respondents, 42%) (Table 6).

**Table 6:** Providers’ reasons for not using cocaine. Many respondents gave multiple reasons

Reason	Number of respondents (%)
Equivalent decongestants available	46 (65%)
Availability	30 (42%)
Medicolegal risk	22 (31%)
Implications of narcotic use	17 (24%)
Other	14 (20%)
Cost	4 (5.6%)

Sixty respondents (63%) used topical epinephrine for decongestion in ESS. Six respondents (6%) used Moffett’s Solution (cocaine + epinephrine), including 3 respondents (3%) who used both, giving a total of 63 respondents (66%) who used an epinephrine preparation in ESS (Table 1).

Reported usage among the respondents is given in Table 7. Physicians who had been in practice for less than 10 years were more likely to use epinephrine than those who had been in practice for more than 10 years (79% vs. 53% [*P =* .015]) (Table 7). Epinephrine usage also differed by province with the highest rates in Newfoundland, New Brunswick (3 of 3 respondents each), Nova Scotia and Prince Edward Island (2 of 2 respondents each). The lowest rate was in British Columbia (2 of 10 respondents, 17%).

**Table 7:** Use of Cocaine and Epinephrine by number of years in practice

	Years in Practice		*P* Value
	< 10 years: n, (%)	≥ 10 years: n, (%)	
Respondents who use cocaine for ESS (any preparation)	4 (12%)	19 (32%)	0.037*
Respondents who use epinephrine for ESS (any preparation)	26 (79%)	32 (53%)	0.015*

* denotes statistical significance

Among the 60 respondents that utilized isolated topical epinephrine, the most common concentration used was 1:1000, with 53 respondents (88%).

For locally injected decongestant, 85 respondents (88%) use epinephrine and lidocaine and 5 respondents (5%) use isolated epinephrine. The most popular concentration of injected epinephrine is 1:100000 (68 respondents, 71%), followed by 1:200000 (16 respondents, 17%). Eighty-four respondents (88%) inject trans-nasally and 10 respondents (10%) inject trans-orally, including 9 respondents who do both (Table 8). Four respondents (4%) do not inject local decongestant in sinus surgery. The most common locations for injection are the lateral wall (67 respondents, 70%) and the middle turbinate head (67 respondents, 70%).

**Table 8:** Structures targeted by respondents for transnasal injection of local decongestant in ESS. Respondents chose multiple structures

Structure	Number of Respondents	Proportion of Respondents (%)
Lateral wall	67	69.8%
Axilla	45	46.9%
Middle Turbinate Head	67	69.8%
Sphenopalatine Region	20	20.8%
Face of Sphenoid	9	9.4%
Other	17	17.7%

When asked about the factors that influenced practitioners’ choice of topical decongestant technique, 62 respondents (65%) reported their residency as an important factor and 19 respondents (20%) reported their fellowship training as an important factor. Evidence from the literature was a factor for 25 respondents (26%) (Table 9).

**Table 9:** Factors influencing respondents’ choice of topical decongestant technique for ESS

Factor	Number of Respondents	Proportion of Respondents (%)
Residency	62	64.6%
Literature	25	26%
Fellowship	19	19.8%
Medicolegal Liability	15	15.6%
Other	12	12.5%
Safety	7	7.3%
Cost	5	5.2%

Other than epinephrine, the most commonly used decongestant was Xylometazoline (44 respondents, 46%), followed by Oxymetazoline (35 respondents, 36%) (Table 1).

## Discussion

This is the first investigation of Canadian otolaryngologists’ practices for nasal decongestion in ESS. Over the years there has been a trend of declining use of cocaine for nasal decongestion. In the United States, Long et al. repeated methodology from a 1977 survey 25 years later and found that cocaine use had significantly decreased, with only 65% having used it in the last 10 years compared to 92% in 1977 [7]. In our study, only 27% of respondents report using some form of cocaine (isolated or Moffett’s Solution) in their current practice for ESS. As in our study, Long et al. found that otolaryngologists who were in practice for less than 10 years were significantly less likely to use cocaine than those in practice for longer.

Compared to the most recent data from other nations, the prevalence of cocaine administration among Canadian otolaryngologists’ (27%) is the lowest yet reported. A 2002 survey in Australia found 64% of otolaryngologists used cocaine in nasal surgery [1]. A 2011 study in the United Kingdom found that 68% of British otolaryngologists used a topical cocaine solution regularly for decongestion [9]. A 2004 study from the United States found that 50% had used cocaine for nasal surgery in the past year [7].

The most common reason for not using cocaine in ESS was providers’ satisfaction with other available decongestants. The next most common reason for avoiding cocaine was restrictions in availability, including a lack of medical-grade cocaine suppliers in some cities in Canada.

The safety of cocaine when used as recommended in nasal surgery has not been well investigated. The most common reported complications include arrhythmia, hypertension and cardiac arrest. Mortality is rare: in a survey of 360 otolaryngologists with a combined 3417 total practice-years, only one mortality possibly related to cocaine was reported [8]. In our study of 96 otolaryngologists, two mortalities possibly related to cocaine were reported over a total of over 1500 practice-years. The generally accepted maximum safe dose of cocaine is 200 mg, which is based on a 1923 study of 50 high-dose cocaine-related deaths [10], but adverse events have occurred at a lower doses. In this survey, all of the otolaryngologists who use cocaine and reported adverse events do not use doses above 200 mg. In total, 12% of respondents report personally experiencing an adverse event that could be attributed to cocaine use, including temporary arrhythmias, temporary hypertension, allergic reactions and cardiac arrest. Notably, the most severe adverse reactions were witnessed by those respondents who do not use cocaine in ESS, and it is possible that their past experiences play a role in their decision to avoid it. Among the 6 respondents who use Moffett’s solution, no adverse events related to cocaine were reported.

Interestingly, one of the reported events was in a patient who abused cocaine pre-operatively, and had a cardiac arrest after xylometazoline was administered. This serves as a reminder of the importance of screening for cocaine abuse before surgery, regardless of the decongestant used. Fifty-six percent of providers who use cocaine for ESS take into account patient comorbidities in their decision to use the drug. The factors that deter providers from using cocaine in certain patients include allergy, arrhythmia, hypertension, coronary artery disease, and a history of drug abuse. It is possible that our study overestimates the frequency of adverse events related to cocaine, as those who have had negative experiences may be more likely to respond to a survey about intraoperative cocaine use.

In similar studies of otolaryngologists in other nations, a combination of cocaine and epinephrine (Moffett’s Solution) has proved popular, with a recent U.K. study reporting that 53% of respondents used it for nasal surgery [9]. There is some evidence that co-administration of epinephrine and cocaine increases effectiveness and safety. However, only 6% of respondents in this study used this preparation. Interestingly, none of these respondents reported witnessing adverse events related to cocaine.

Although our response rate of 19% was less that most previous studies on this topic, it represents an above-average response rate for electronic surveys sent out to CSO-HNS members. The low completion rate may be partially due to the survey having no opt-out mechanism for respondents who have other sub-specialty expertise or do not perform sinus surgery.

Our question “which of the following solutions do you use for decongestion of nasal mucosa?” captures current practices by respondents. Our results show that an unexpectedly low proportion of Canadian otolaryngologists currently use cocaine for ESS. To elucidate why this finding is different from previous studies, practice pattern surveys in the near future could be performed in other jurisdictions, especially the United States, the United Kingdom, and Australia, where previous data exists to see if a global shift in decongestant practices is present.

## Conclusion

This study shows that the decongestant practices of Canadian otolaryngologists for ESS differ significantly from those reported in other nations and in the past. Notably, the use of cocaine for decongestion is much lower than previous studies in other countries. Most respondents’ experience with cocaine did not include adverse events with long-term consequences, and use of cocaine with epinephrine (Moffett’s Solution) was not associated with any adverse events. Many practitioners have adopted the use of 1:1000 epinephrine, citing equivalency to cocaine with an improved safety profile as the primary reason. Regardless of the decongestant used, providers should make their choice based on patient factors.

## Additional files

Additional file 1: Complete Online Survey - English version. (DOCX 25 kb)

Additional file 2: Complete Online Survey - French version. (DOCX 27 kb)
