# Supplementary material for: Contemporary decongestant practices of Canadian otolaryngologists for endoscopic sinus surgery
Source: J Otolaryngol Head Neck Surg. 2019 Mar 18;48:15. doi: 10.1186/s40463-019-0337-8 (PMC6421656; doi:10.1186/s40463-019-0337-8)
Supplement: Supplementary file 1 — Complete Online Survey - English version. (DOCX 25 kb) [file 40463_2019_337_MOESM1_ESM.docx]

Use of cocaine in sinus surgery

Start of Block: Block1

Q1 *This survey is available in both French and English, make your selection above.
 Ce sondage est disponible en français et en anglais, veuillez sélectionner au-dessus.*


 **Project Title:** Use of cocaine in sinus surgery

**Principal Investigator**: Dr. Leigh Sowerby, MD, FRCSC, Department of Otolaryngology–Head & Neck Surgery, Schulich School of Medicine and Dentistry.

**Letter of Information**

**1. Invitation to Participate**

You are being invited to participate in this research study about which decongestant medications otolaryngologists use for Endoscopic Sinus Surgery, and whether they have experienced any adverse events with these drugs, because you belong to the Canadian Society of Otolaryngology-Head and Neck Surgery (CSO-HNS).

**2. Purpose of the Letter**

The purpose of this letter is to provide you with information required for you to make an informed decision regarding participation in this research.

**3. Background/ Purpose of this Study**

Common drugs for decongestion in Endoscopic Sinus Surgery include cocaine, phenylephrine, oxymetazoline, and epinephrine. Our understanding of the safety of each agent is changing, as are the practices of Otolaryngologists-Head & Neck Surgeons. Canadian practices regarding decongestion in Endoscopic Sinus Surgery have never been investigated. Our objective is to determine the decongestion practices in FESS across Canada. The results will help determine the trends in this field, and may identify factors that influence these practices. This may help establish reasonable standards of care for decongestant practices, and will allow us to compare our practices with other nations.

**4. Inclusion Criteria**

Individuals who are active members of the Canadian Society of Otolaryngology-Head and Neck Surgery, and are practicing staff surgeons, are eligible to participate in this study.

**5. Exclusion Criteria**

Individuals who are in the residency or fellowship stage of their training, or those who are retired from practice, are not eligible to participate in this study.

**6. Study Procedures**

If you agree to participate, you will be asked to complete an online survey about what decongestant techniques you use for nasal surgery. It is anticipated that the entire task will take five minutes, one time. There will be a total of roughly 200 participants.

**7. Possible Risks and Harms**

Some of the survey questions ask about adverse effects you’ve observed during sinus surgery and may be distressing to you as you think about your experiences.

**8. Possible Benefits**

You will receive no direct benefits from participating in this research study. However, your responses may help us learn more about trends in decongestant use among Canadian otolaryngologists for sinus surgery, as well as the safety of common decongestant drugs used for nasal surgery.

**9. Compensation**

You will not be compensated for your participation in this research.

**10. Voluntary Participation**

Participation in this study is voluntary. You may refuse to participate, refuse to answer any questions or withdraw from the study at any time with no effect on your future academic status and no effect on your employment.

**11. Confidentiality**

Your survey answers will be sent to a link at Qualtrics.com, an anonymous online survey platform, where data will be stored in a password protected electronic format. Qualtrics does not collect identifying information such as your name, email address, or IP address. Therefore, your responses will remain anonymous. No one will be able to identify you or your answers, and no one will know whether or not you participated in the study. All data collected will remain confidential and accessible only to the investigators of this study. All data will be stored on a password protected encrypted database on a hospital computer and stored on a secure (S:) network drive behind the hospital firewall for 15 years. After this, all data will be destroyed. As each survey response is anonymous, individual survey responses cannot be identified and deleted. If the results are published, your email address will not be used. While we will do our best to protect your information there is no guarantee that we will be able to do so. Representatives of The University of Western Ontario Health Science Research Ethics Board may contact you or require access to your study-related records to monitor the conduct of the research.

**12. Contacts for Further Information**

If you have questions at any time about the study or the procedures, you may contact my research supervisor, Dr Leigh Sowerby via email at Leigh.Sowerby@sjhc.london.on.ca.    If you have any questions about your rights as a research participant or the conduct of this study, you may contact The Office of Research Ethics (519) 661-3036, email: ethics@uwo.ca.    If you have other questions, comments or complaints, you can reach the Patient Experience Coordinator at 519-685-8500 ext. 52036.

**13. Publication**

If the results of the study are published, your name will not be used. If you would like to receive a copy of any potential study results, please contact Dr Leigh Sowerby at Leigh.Sowerby@sjhc.london.on.ca.

**14. Consent**

Completion of the survey is indication of your consent to participate.

- Agree
- Disagree

Q2 What is your current status?

- Active practice
- Retired/Resident/Fellow

Q3 How many years have you been in practice?

Q4 In which province are you currently practicing/training?

- Alberta
- British Columbia
- Manitoba
- New Brunswick
- Newfoundland/Labrador
- Northwest Territories
- Nova Scotia
- Nunavut
- Ontario
- Prince Edward Island
- Quebec
- Saskatchewan
- Yukon
- International

Q6 Where did you receive your otolaryngology residency training?

- Alberta
- British Columbia
- Manitoba
- Nova Scotia
- Ontario
- Quebec
- Saskatchewan
- USA
- Australia
- Europe
- Asia
- Other

Q25 Where did you receive your otolaryngology fellowship training?

- Alberta
- British Columbia
- Manitoba
- Nova Scotia
- Ontario
- Quebec
- Saskatchewan
- USA
- Australia
- Europe
- Asia
- Other

Q8 In which of the following settings are you currently practicing/training? (Check all that apply)

- Academic Practice
- Private Practice

Q9 How much of your practice is devoted to sinonasal inflammatory disease?

- 0-25%
- 25-50%
- 50-75%
- 75%-100%

Q10 In what subspecialty are you fellowship trained? (Check all that apply)

- Nil/General
- Head and Neck
- Facial Plastics
- Rhinology
- Laryngology
- Otology
- Pediatrics
- Other sub-specialty training (please specify) ________________________________________________

Q12
For the following questions, please consider them in the context of performing endoscopic sinus surgery for any variant of chronic sinusitis.


Please choose which of the following solutions you use for decongestion of nasal mucosa (Check all that apply):

- Cocaine
- Epinephrine
- Phenylephrine
- Moffett’s Solution (Cocaine and Epinephrine)
- Xylometazoline
- Oxymetazoline
- Other (Please specify) ________________________________________________

Q13 Which formulation of Cocaine do you use (Check all that apply)

- - 4% solution
- - 10% solution
- - 20% paste
- - Other: (please specify) ________________________________________________

Q14 Which formulation of topical Epinephrine do you use (Check all that apply)?

- - 1:1000 solution – topical
- - 1:10,000 solution
- - Other (please specify) ________________________________________________

Q15 Which formulation of Moffett’s solution do you use (Check all that apply)?

- - 1.2% cocaine in 1:10,000 epinephrine solution
- - 4% cocaine in 1:1000 epinephrine solution
- - 4% cocaine in 1:10,000 epinephrine solution
- - 10% cocaine in 1:10,000 epinephrine solution
- - Other: (please specify) ________________________________________________

Q16 Do you try to keep the maximum dose of cocaine to below 200mg?

- - Yes
- - No
- - Unsure

Q17
Does a patient’s medical comorbidities affect your choice to use cocaine?

- - Yes (Explain:) ________________________________________________
- - No

Q18
Do you use cocaine in pediatric (<12 years old) patients?

- - Yes
- - No

Q19 If you do not use cocaine, why do you not do so (select all that apply)?

- - Cost
- - Availability
- - Implications of narcotic usage
- - Medicolegal risk
- - Equivalent decongestants available
- - Other: ________________________________________________

Q20 Which forms of Injected decongestant do you use (Check all that apply)?

- - Epinephrine and Lidocaine
- - Epinephrine alone
- - Other (please specify) ________________________________________________

Q21 Which technique of injection do you use?

- - Trans-nasal
- - Trans-oral (Sphenopalatine injection)
- - Other (please specify) ________________________________________________

Q22
Which structures do you inject trans-nasally? (Check all that apply)

- - Lateral wall
- - Axilla
- - Middle turbinate head
- - Sphenopalatine region
- - Face of sphenoid
- - Other (please specify) ________________________________________________

Q26 Which concentration of epinephrine do you use?

- 1:100 000
- 1:200 000
- Other (please specify) ________________________________________________

Q23
What factors do you consider in determining your choice of topical decongestant technique (Select all that apply)?

- - Residency training
- - Fellowship training
- - Medicolegal liability
- - Recent literature
- - Cost
- Other: (please specify) ________________________________________________

Q24
Have you PERSONALLY ever had a patient experience an intraoperative cardiovascular event during endoscopic sinus surgery?

- - Yes (please explain outcome and factors influencing events, including medication and concentrations used (maintaining patient confidentiality)) ________________________________________________
- - No

Q25
Have you PERSONALLY ever had a patient experience any intraoperative or postoperative adverse event that could be attributable to usage of cocaine?

- - Yes (please explain outcome and factors influencing events (maintaining patient confidentiality)) ________________________________________________
- - No, I use cocaine intraoperatively and have never had any adverse events as a result
- - No, I do not use cocaine intraoperatively

Q26 Any comments?

________________________________________________________________
