# Supplementary material for: Contemporary decongestant practices of Canadian otolaryngologists for endoscopic sinus surgery
Source: J Otolaryngol Head Neck Surg. 2019 Mar 18;48:15. doi: 10.1186/s40463-019-0337-8 (PMC6421656; doi:10.1186/s40463-019-0337-8)
Supplement: Supplementary file 2 — Complete Online Survey - French version. (DOCX 27 kb) [file 40463_2019_337_MOESM2_ESM.docx]

Use_of_cocaine_in_sinus_surgery

Start of Block: Block1

Q1
 **Nom du projet :**

Utilisage de la cocaïne dans la chirurgie des sinus.

**Chercheur Principal :** Dr. Leigh Sowerby, MD, FRCSC, Départmente de l’Otolaryngology–Head & Neck Surgery, Schulich School of Medicine and Dentistry.

**Document d’information**

1. **Invitation à participer**

Parce que vous êtes un membre de Canadian Society of Otolaryngology-Head and Neck Surgery (CSO-HNS), vous êtes invités à participer à cette sondage au sujet des médicaments que vous utilisez pour la chirurgie endoscopique des sinus, et si vous avez expérience des événements adverses avec ces médicaments.

1. **L’objectif du document**

L’objectif du document est de vous donner de l’information essentielle pour faire une décision informée concernant votre participation en ces recherches.

1. **Description/Objectif de la recherche**

Quelques décongestionnants habituels pour la chirurgie endoscopique des sinus sont la cocaïne, la phényléphrine, l’oxymétazoline, et l‘épinéphrine. Notre compréhension de la sécurité de ces médicaments est dans un état d’avancement, comme avec les pratiques des otolaryngologistes. Les pratiques des chirurgiens canadiens concernant les décongestionnants dans la chirurgie endoscopique des sinus n’étaient jamais étudiées. Notre objectif est de déterminer ces pratiques à travers le Canada. Les résultats peuvent s’aider à établir des normes de soins dans ce domaine, et comparer nos pratiques avec l’eux des autres pays.

1. **Critères d'inclusion**

Des personnes qui sont des membres actifs de la Canadian Society of Otolaryngology-Head and Neck Surgery, et qui sont des chirurgiens consultants, sont éligibles à participer en cette sondage.

1. **Critères d'exclusion**

Des personnes qui sont des résidents ou des moniteurs, ou des chirurgiens retirés, ne sont pas éligibles à participer en ce sondage.

1. **Procédures de la recherche**

Si vous acceptez de participer, vous serez demandés à compléter un sondage concernant vos pratiques pour décongestionner le nez pour la chirurgie endoscopique des sinus. Le sondage prend cinq minutes, et c’est juste une fois. En total, il y aura presque 200 participants.

1. **Risques et dommages possibles**

Il y a quelques questions au sujet des effets adverses que vous avez observé pendant la chirurgie. Elles peuvent possiblement être stressant pour vous.

1. **Bienfaits possibles**

Vous n’allez pas recevoir des bienfaits directs pour votre participation à cette sondage. Toutefois, vos réponses peuvent nous aider à apprendre plus au sujet de l’utilisage des décongestionnants parmi les otorhinolaryngologistes canadiennes, et la sécurité des décongestionnants communs.

1. **Compensation**

Vous n’allez pas recevoir de la rémunération pour votre participation en ce sondage.

1. **Participation volontaire**

Votre participation à cette sondage est volontaire. Vous pouvez refuser de participer ou discontinuer le sondage n’importe quand sans pénalité avec aucun effet sur votre statut académique ou votre occupation.

1. **Confidentialité**

On va envoyer vos réponses à Qualtrics.com, un service électronique pour les sondages anonymes. Là, les données vont être protégées sous mot de passe. Qualtrics ne vont pas collectionner des données personnelles comme votre nom, adresse de courriel, ou adresse IP. Donc, vos réponses seront anonymes. Personne ne pourra vous identifier, et personne ne va savoir si vous avez participé à cette sondage. Tous les données vont rester confidentielles et accessibles seulement par les chercheurs de ce sondage. Tous les données vont être enregistrées dans un réseau sécure derrière un pare-feu pour 15 ans. Après ça, tous les données vont être détruites. Parce que chaque réponse est anonyme, les réponses individuelles ne peuvent pas être identifiées et supprimées. Si les résultats sont publiés, votre courriel ne vont pas être utilisé. Même si on fait son mieux, il n’y a pas garanti qu’on peut protéger vos infos. C’est possible que les représentatifs du comité d'éthique de la recherche de la University of Western Ontario vous contacterez ou aurez besoin de l’accès à vos infos concernant ce sondage pour monitorer le conduit de la recherche.

1. **Nous Joindre**

Si vous avez des questions au sujet du sondage, vous pouvez joindre mon superviseur, Dr Leigh Sowerby par courriel : Leigh.Sowerby@sjhc.london.on.ca. Si vous avez des questions concernant vos droits comme participant, ou concernant le conduit du sondage, vous pouvez contacter : The Office of Research Ethics (519) 661-3036, courriel: ethics@uwo.ca Si vous avez des autres questions, des plaintes, ou des concernes, le Patient Experience Coordinator à 519-685-8500 ext. 52036.

1. **Publication**

Si les résultats sont publiés, votre courriel ne vont pas être utilisé. Si vous voulez recevoir une copie des résultats, veuillez contacter Dr Leigh Sowerby à Leigh.Sowerby@sjhc.london.on.ca.

1. **Accord**

Si vous complétez le sondage, ça indique que vous donnez votre accord à participer.

- D'accord (1)
- Pas d'accord (2)

Q2 C’est quoi votre état?

- Pratiquer en courant (1)
- Retiré/Résident/Moniteur (2)

Q3 Vous avez combien d’ans en pratique?

Q4
Dans quelle province est-ce que vous pratiquez?

- Alberta (1)
- British Columbia (2)
- Manitoba (3)
- New Brunswick (4)
- Newfoundland/Labrador (5)
- Northwest Territories (6)
- Nova Scotia (7)
- Nunavut (8)
- Ontario (9)
- Prince Edward Island (10)
- Quebec (11)
- Saskatchewan (12)
- Yukon (13)
- International (14)

Q6
Où avez-vous faites votre résidence?

- Alberta (1)
- British Columbia (2)
- Manitoba (3)
- Nova Scotia (7)
- Ontario (9)
- Quebec (11)
- Saskatchewan (12)
- USA (14)
- Australia (13)
- Europe (10)
- Asia (8)
- Other (6)

Q25
Où avez-vous faites votre stage de perfectionnement?

- Alberta (1)
- British Columbia (2)
- Manitoba (3)
- Nova Scotia (7)
- Ontario (9)
- Quebec (11)
- Saskatchewan (12)
- USA (14)
- Australia (13)
- Europe (10)
- Asia (8)
- Other (6)

Q8 Dans quelles milieus est-ce que vous pratiquez actuellement?

- Académie (1)
- Privée (2)

Q9 Quelle proportion de votre pratique est dévouée aux maladies du sinus et nez?

- 0-25% (1)
- 25-50% (2)
- 50-75% (3)
- 75%-100% (4)

Q10 Lesquelles sont les sous-spécialités en quelles vous avez fait une programme de formation complémentaire?

- Aucun/general (1)
- Tête et cou (2)
- Chiurgie plastique du visage (3)
- La rhinologie (4)
- La laryngologie (5)
- L’otologie (6)
- La pédiatrie (7)
- Autre (Spécifiez s’il-vous-plaît) (8) ________________________________________________

Q12

Veuillez considerez les prochaines questions dans la contexte de la chirurgie endoscopique des sinus pour n’importe quelle type du sinusite chronique:


Veuillez sélectionner toutes les solutions que vous utilisez pour décongestionner le nez.

- Cocaïne (1)
- épinéphrine (2)
- Phenylephrine (3)
- Moffett’s Solution (Cocaïne et épinéphrine) (4)
- Xylometazoline (5)
- oxymetazoline (6)
- Autre (Veuillez spécifier) (7) ________________________________________________

Q13
Quelles formulations de cocaïne est-ce que vous utilisez?

- - 4% solution (1)
- - 10% solution (2)
- - 20% pâte (3)
- - Autre (Veuillez spécifier) (4) ________________________________________________

Q14
Quelles formulations d’épinéphrine topicale est-ce que vous utilisez?

- - 1:1000 solution – topical (1)
- - 1:10,000 solution (2)
- - Autre (Veuillez spécifier) (3) ________________________________________________

Q15
Quelle formulation de la solution de Moffett est-ce que vous utilisez?

- - 1.2% cocaïne et 1:10,000 épinéphrine en solution (1)
- - 4% cocaïne et 1:1000 épinéphrine en solution (2)
- - 4% cocaïne et 1:10, 000 épinéphrine en solution (3)
- - 10% cocaïne et 1:10, 000 épinéphrine en solution (4)
- - Autre (Veuillez spécifier) (5) ________________________________________________

Q16
Est-ce que vous attemptez d’utilisez moins de 200mg en total de cocaïne?

- - Oui (1)
- - Non (2)
- - Je ne sais pas (3)

Q17
Est-ce que les comorbidités d’un patient inflencent votre decision d’utiliser le cocaine? 

- - Oui (Veuillez Expliquer) (1) ________________________________________________
- - Non (2)

Q18
Est-ce que vous utilisez le cocaïne pour les patients pediatriques (<12 ans)?

- - Oui (1)
- - Non (2)

Q19
Si vous n’utilisez pas le cocaïne, pourquoi est-xe que vous ne l’utilisez pas (sélectionnez tous les réponses qui s’appliquent) ?

- - Coût (1)
- - disponibilité (2)
- - les implications d’utiliser les narcotiques (3)
- - La risqué médicolégal (4)
- - Disponibilité des décongestionnants egales (5)
- - Autre: (Veuillez specifier) (6) ________________________________________________

Q20
Quelles types de décongestionnants injectées est-ce que vous utilisez?

- - épinéphrine et Lidocaïne (1)
- - épinéphrine tout seul (2)
- - Autre: (Veuillez specifier) (3) ________________________________________________

Q21 Quelle technique d’injection est-ce que vous utilisez?

- - Trans-nasale (1)
- - Trans-orale (sphenopalatine) (2)
- - Autre (Veuillez spécifiez) (3) ________________________________________________

Q22
a) Quelles structures est-ce que vous injectez par le nez?

- - le mur latéral (1)
- - l'Axilla (2)
- - Tête du cornet moyen (3)
- - La region sphenopalatine (4)
- - La face de le sphenoid (5)
- - Autre (veuillez spécifiez) (6) ________________________________________________

Q26 Quelles formulations d’épinéphrine est-ce que vous utilisez?

- 1:100 000 (1)
- 1:200 000 (2)
- Other (please specify) (3) ________________________________________________

Q23
Quelles facteurs est-ce que vous considérez en déterminant votre choix de décongestionnant topicale?

- - Résidence (1)
- - Stage de perfectionnement (2)
- - Liabilité médico-légal (3)
- - Literature récente (4)
- - Coût (5)
- - Autre: (Veuillez spécifiez) (6) ________________________________________________

Q24
Est-ce que vous avez jamais vous-même eu un patient qui a experiencé un évenement cardiovasculaire pendant la chirurgie sinus endoscopique?

- - Oui (veuillez expliquer le résultat et les facteurs qui l’a influencé, comme les medicaments et concentrations utilises, sans violer la confidentialité) (1) ________________________________________________
- - Non (2)

Q25
Est-ce que vous avez jamais vous-même eu un patient qui a experiencé n’importe quelle sorte d’évenement advers pendant ou après la chirurgie sinus endoscopique, qui peut être attribué à la cocaïne?

- - Oui (veuillez expliquer le résultat et les facteurs qui l’a influencé, comme les medicaments et concentrations utilises, sans violer la confidentialité) (1) ________________________________________________
- - Non, j’utilise la cocaïne dans les chirurgies et je n’ai jamais expériencé des événements adverses comme résultat (2)
- Non, je n’utilise pas la cocaïne dans les chirurgies (3)

Q26 Si vous avez des commentaires, mettez-les içi:

________________________________________________________________
